# Biotinidase deficiency: A treatable cause of infantile seizures

**DOI:** 10.4103/1817-1745.66660

**Published:** 2010

**Authors:** Parveen Bhardwaj, Ram Krishan Kaushal, Akshat Chandel

**Affiliations:** Department of Pediatrics, Indira Gandhi Medical College, Shimla, HP, India

Sir,

Biotinidase deficiency is a rare metabolic disorder with an estimated incidence of 1:61,067 population, although severe or profound disease is much rarer (1:1,37,401 population).[1]

Clinical manifestations include neurological, dermatological, immunological and ophthalmological abnormalities.[2] Biochemically, the disease is characterized by metabolic acidosis and organic aciduria. Treatment with biotin results in pronounced, rapid, clinical and biochemical improvement, but some patients have residual neurological damage comprising neurosensory hearing loss, visual pathway defects, ataxia and mental retardation.[3]

**Figure 1:**
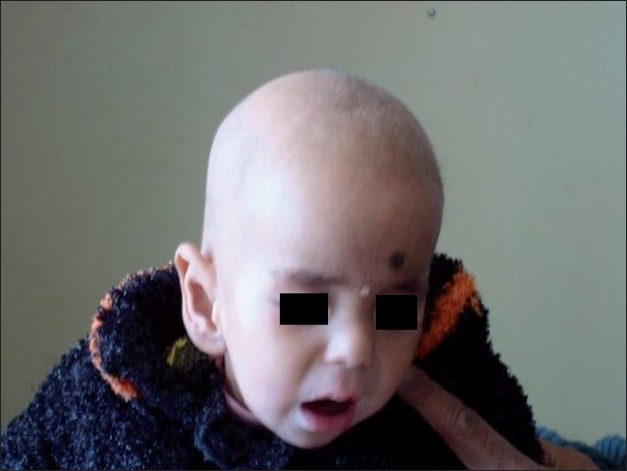
Alopecia in biotinidase deficiency

A 6-month-old male child born of nonconsanguineous marriage presented with a history of seizures from 3 months of age and was being treated with sodium valproate, but seizure control was not observed. Antenatal and natal history was uneventful. There was no family history of seizure disorders. According to the mother, the child developed normally for the first 3 months and after that was not growing normally. On examination, the child had normal neurological findings, except for hypotonia, irritability and alopecia [Figure 1]. The child also had a mild developmental delay. Laboratory investigations revealed normal hemogram, liver functions, serum ammonia and serum electrolytes. Blood gas analysis showed metabolic acidosis and the baby also had persistent ketonuria, while cerebrospinal fluid examination was normal. Ophthalmological and brainstem-evoked audiometry examination was normal and magnetic resonance imaging revealed diffuse cortical atrophy. A specific enzyme assay showed deficient biotinidase activity of 0.6 nmol/min/mL (normal >5 nmol/min/mL). The child was given biotin (20 mg/day) orally. This treatment produced a pronounced, rapid, clinical and biochemical improvement and good control over seizures, resulting in discontinuation of antiepileptic drugs.

Biotin is a cofactor required by acetyl CoA carboxylase, pyruvate carboxylase, propionyl CoA carboxylase and 3 methylcrotonyl CoA carboxylase. It is covalently attached to the apocarboxylases by the epsilon amino group of a lysine residue, where it functions at the active site as a carbon dioxide carrier in the carboxylation reactions. Biotinylation of the apocarboxylases is catalyzed by holocarboxylase synthetase in an adenosine triphosphate-dependent reaction, with the intermediate formation of biotinyl adenosine monophosphate. The turnover of carboxylases yields biotinyllysine (biocytin) from which biotin is regenerated by the action of a specific amidolyase, biotinidase. This enzyme is also required for the release of dietary protein-bound biotin.[3]

Individual inherited disorders of all four biotin-dependent carboxylases have been reported in addition to reports of patients with simultaneous defects of all four enzymes (combined carboxylase deficiency). In a majority of patients with late-onset combined carboxylase deficiency, the underlying defect is biotinidase deficiency.[4] The early-onset form, on the other hand, is due to holocarboxylase synthetase deficiency.[5]

Biotinidase deficiency can be profound (<10% enzyme level) or partial (10-30% enzyme level). Clinical presentation depends on the severity of enzymatic defect. Profound defects usually manifest between 3 and 6 months of age, with neurological manifestations (seizures, hypotonia and developmental delay), skin manifestations (eczematous skin rash, seborrheic dermatitis, alopecia) and (c) respiratory problems (hyperventilation, laryngeal stridor and apnea). Older children and adolescents may exhibit limb weakness, neurosensory hearing loss and eye problems, e.g. optic atrophy and scotomas. The present case had only seizures and alopecia. Many symptomatic children with biotinidase deficiency exhibit various neuroimaging abnormalities, e.g. cerebral edema, attenuated white matter signal, cerebral atrophy and compensatory ventricular enlargement. Neuroimaging features may improve or become normal after biotin treatment.[6]

Laboratory findings include metabolic acidosis and abnormal organic acids in the urine, and diagnosis can be established by estimating biotidinase in the serum. Biotinidase deficiency may be detected on screening of the newborn.[7]

Biotinidase deficiency may be confused with holocarboxylase deficiency, previously called early-onset or infantile multiple or combined carboxylase deficiency, which presents early and the biotidinase level is normal.

Because biotinidase deficiency can be treated readily with biotin, this disorder should be considered in children with infantile seizures, especially in the presence of other characteristic neurological or cutaneous features.
